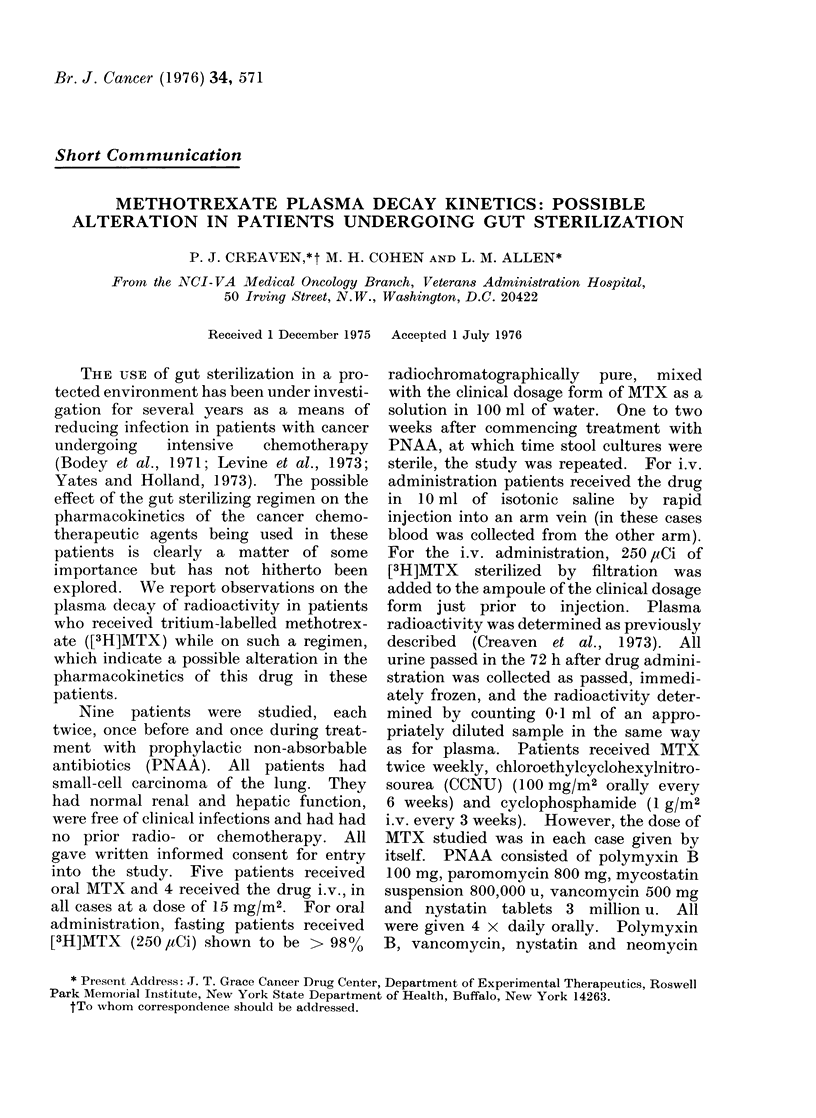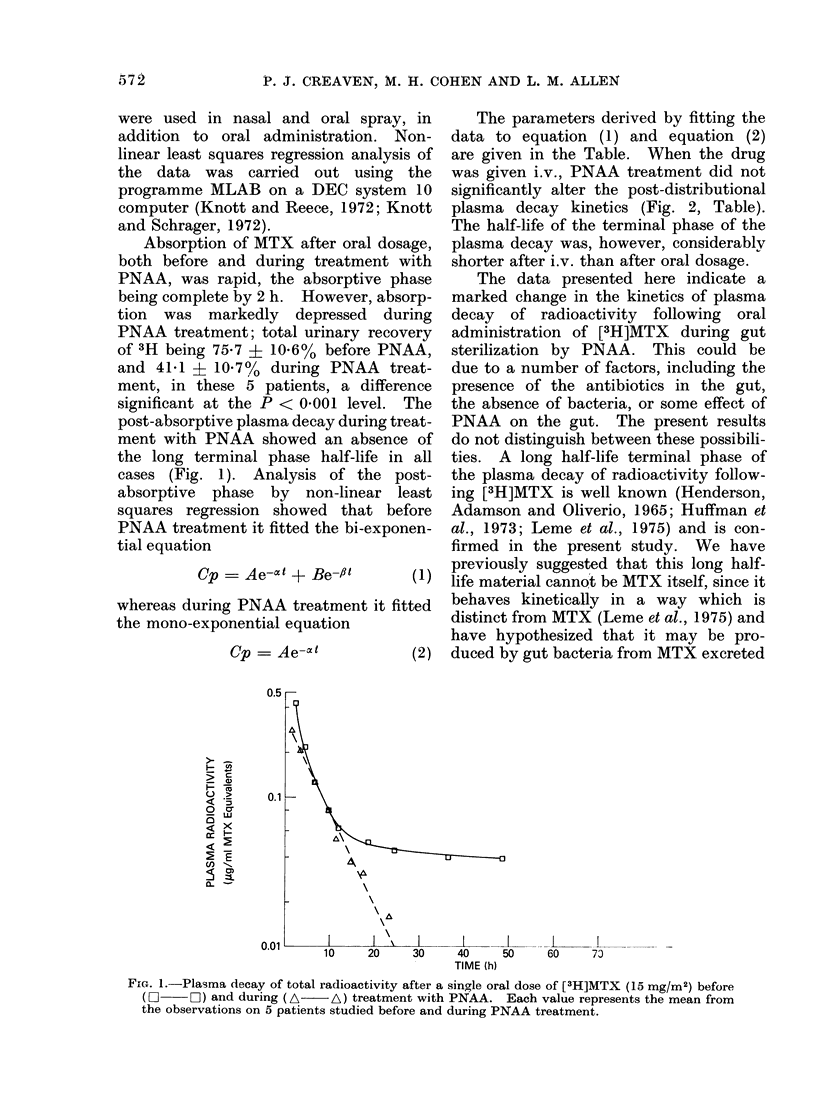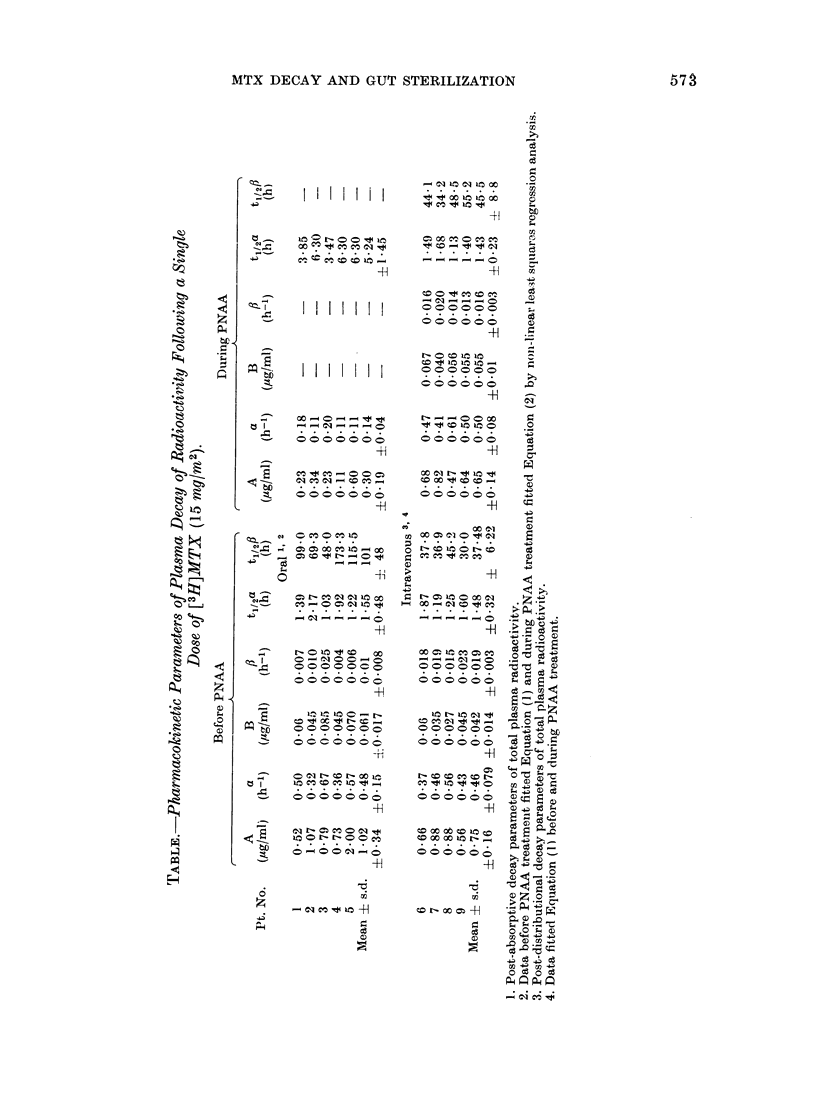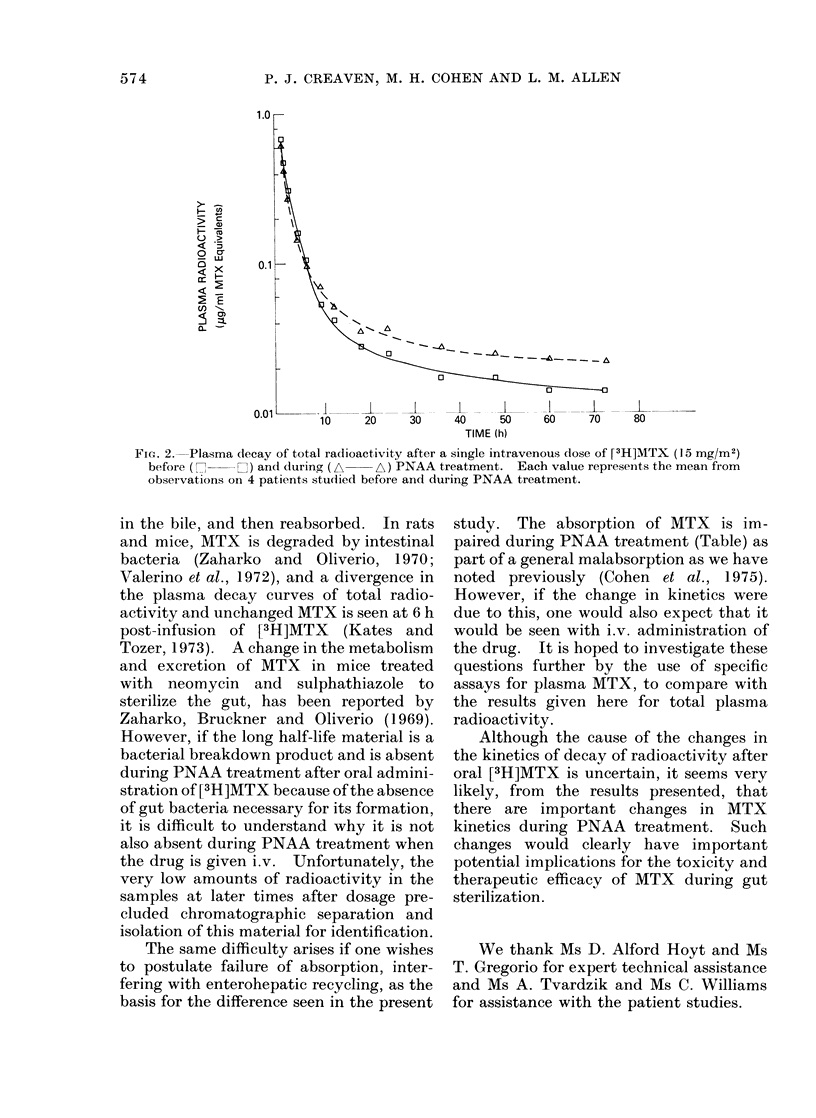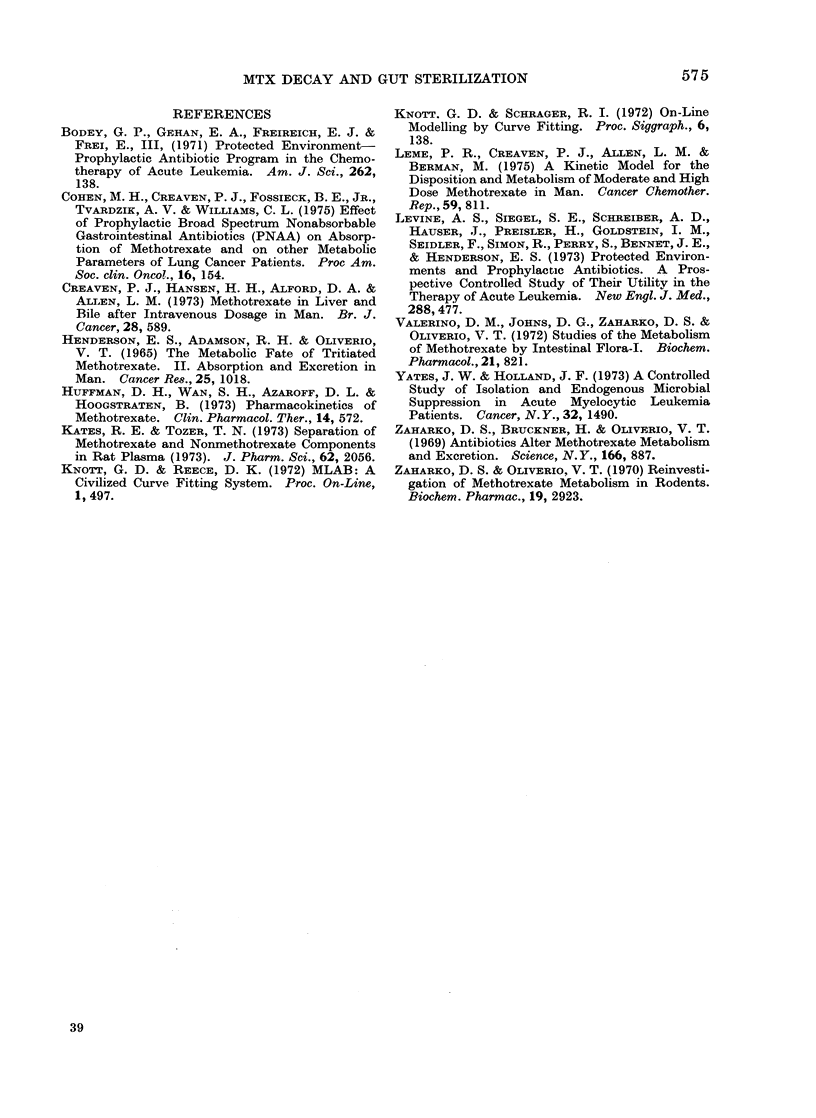# Methotrexate plasma decay kinetics: possible alteration in patients undergoing gut sterilization.

**DOI:** 10.1038/bjc.1976.213

**Published:** 1976-11

**Authors:** P. J. Creaven, M. H. Cohen, L. M. Allen


*Br. J. Cancer* (1976) **34**, 571

***Short Communication***

### METHOTREXATE PLASMA DECAY KINETICS: POSSIBLE ALTERATION IN PATIENTS UNDERGOING GUT STERILIZATION

P. J. CREAVEN,*† M. H. COHEN AND L. M. ALLEN*

*From the NCI-VA Medical Oncology Branch, Veterans Administration Hospital, 50 Irving Street, N.W., Washington, D.C.* 20422

Received 1 December 1975 Accepted 1 July 1976

THE USE of gut sterilization in a protected environment has been under investigation for several years as a means of reducing infection in patients with cancer undergoing intensive chemotherapy (Bodey *et al.*, 1971; Levine *et al.*, 1973; Yates and Holland, 1973). The possible effect of the gut sterilizing regimen on the pharmacokinetics of the cancer chemotherapeutic agents being used in these patients is clearly a matter of some importance but has not hitherto been explored. We report observations on the plasma decay of radioactivity in patients who received tritium-labelled methotrexate ([$^3$H]MTX) while on such a regimen, which indicate a possible alteration in the pharmacokinetics of this drug in these patients.

Nine patients were studied, each twice, once before and once during treatment with prophylactic non-absorbable antibiotics (PNAA). All patients had small-cell carcinoma of the lung. They had normal renal and hepatic function, were free of clinical infections and had had no prior radio- or chemotherapy. All gave written informed consent for entry into the study. Five patients received oral MTX and 4 received the drug i.v., in all cases at a dose of 15 mg/m$^2$. For oral administration, fasting patients received [$^3$H]MTX (250 $\mu$Ci) shown to be $>$ 98% radiochromatographically pure, mixed with the clinical dosage form of MTX as a solution in 100 ml of water. One to two weeks after commencing treatment with PNAA, at which time stool cultures were sterile, the study was repeated. For i.v. administration patients received the drug in 10 ml of isotonic saline by rapid injection into an arm vein (in these cases blood was collected from the other arm). For the i.v. administration, 250 $\mu$Ci of [$^3$H]MTX sterilized by filtration was added to the ampoule of the clinical dosage form just prior to injection. Plasma radioactivity was determined as previously described (Creaven *et al.*, 1973). All urine passed in the 72 h after drug administration was collected as passed, immediately frozen, and the radioactivity determined by counting 0·1 ml of an appropriately diluted sample in the same way as for plasma. Patients received MTX twice weekly, chloroethylcyclohexylnitrosourea (CCNU) (100 mg/m$^2$ orally every 6 weeks) and cyclophosphamide (1 g/m$^2$ i.v. every 3 weeks). However, the dose of MTX studied was in each case given by itself. PNAA consisted of polymyxin B 100 mg, paromomycin 800 mg, mycostatin suspension 800,000 u, vancomycin 500 mg and nystatin tablets 3 million u. All were given 4 × daily orally. Polymyxin B, vancomycin, nystatin and neomycin

* Present Address: J. T. Grace Cancer Drug Center, Department of Experimental Therapeutics, Roswell Park Memorial Institute, New York State Department of Health, Buffalo, New York 14263.

†To whom correspondence should be addressed.

were used in nasal and oral spray, in addition to oral administration. Non-linear least squares regression analysis of the data was carried out using the programme MLAB on a DEC system 10 computer (Knott and Reece, 1972; Knott and Schrager, 1972).

Absorption of MTX after oral dosage, both before and during treatment with PNAA, was rapid, the absorptive phase being complete by 2 h. However, absorption was markedly depressed during PNAA treatment; total urinary recovery of $^3H$ being $75{\cdot}7 \pm 10{\cdot}6\%$ before PNAA, and $41{\cdot}1 \pm 10{\cdot}7\%$ during PNAA treatment, in these 5 patients, a difference significant at the $P < 0{\cdot}001$ level. The post-absorptive plasma decay during treatment with PNAA showed an absence of the long terminal phase half-life in all cases (Fig. 1). Analysis of the post-absorptive phase by non-linear least squares regression showed that before PNAA treatment it fitted the bi-exponential equation

$$Cp = Ae^{-\alpha t} + Be^{-\beta t} \quad (1)$$

whereas during PNAA treatment it fitted the mono-exponential equation

$$Cp = Ae^{-\alpha t} \quad (2)$$

The parameters derived by fitting the data to equation (1) and equation (2) are given in the Table. When the drug was given i.v., PNAA treatment did not significantly alter the post-distributional plasma decay kinetics (Fig. 2, Table). The half-life of the terminal phase of the plasma decay was, however, considerably shorter after i.v. than after oral dosage.

The data presented here indicate a marked change in the kinetics of plasma decay of radioactivity following oral administration of [$^3$H]MTX during gut sterilization by PNAA. This could be due to a number of factors, including the presence of the antibiotics in the gut, the absence of bacteria, or some effect of PNAA on the gut. The present results do not distinguish between these possibilities. A long half-life terminal phase of the plasma decay of radioactivity following [$^3$H]MTX is well known (Henderson, Adamson and Oliverio, 1965; Huffman *et al.*, 1973; Leme *et al.*, 1975) and is confirmed in the present study. We have previously suggested that this long half-life material cannot be MTX itself, since it behaves kinetically in a way which is distinct from MTX (Leme *et al.*, 1975) and have hypothesized that it may be produced by gut bacteria from MTX excreted

FIG. 1.—Plasma decay of total radioactivity after a single oral dose of [$^3$H]MTX (15 mg/m$^2$) before (□——□) and during (△——△) treatment with PNAA. Each value represents the mean from the observations on 5 patients studied before and during PNAA treatment.

FIG. 2.—Plasma decay of total radioactivity after a single intravenous dose of [$^3$H]MTX (15 mg/m$^2$) before (□——□) and during (△——△) PNAA treatment. Each value represents the mean from observations on 4 patients studied before and during PNAA treatment.

in the bile, and then reabsorbed. In rats and mice, MTX is degraded by intestinal bacteria (Zaharko and Oliverio, 1970; Valerino *et al.*, 1972), and a divergence in the plasma decay curves of total radioactivity and unchanged MTX is seen at 6 h post-infusion of [$^3$H]MTX (Kates and Tozer, 1973). A change in the metabolism and excretion of MTX in mice treated with neomycin and sulphathiazole to sterilize the gut, has been reported by Zaharko, Bruckner and Oliverio (1969). However, if the long half-life material is a bacterial breakdown product and is absent during PNAA treatment after oral administration of [$^3$H]MTX because of the absence of gut bacteria necessary for its formation, it is difficult to understand why it is not also absent during PNAA treatment when the drug is given i.v. Unfortunately, the very low amounts of radioactivity in the samples at later times after dosage precluded chromatographic separation and isolation of this material for identification.

The same difficulty arises if one wishes to postulate failure of absorption, interfering with enterohepatic recycling, as the basis for the difference seen in the present study. The absorption of MTX is impaired during PNAA treatment (Table) as part of a general malabsorption as we have noted previously (Cohen *et al.*, 1975). However, if the change in kinetics were due to this, one would also expect that it would be seen with i.v. administration of the drug. It is hoped to investigate these questions further by the use of specific assays for plasma MTX, to compare with the results given here for total plasma radioactivity.

Although the cause of the changes in the kinetics of decay of radioactivity after oral [$^3$H]MTX is uncertain, it seems very likely, from the results presented, that there are important changes in MTX kinetics during PNAA treatment. Such changes would clearly have important potential implications for the toxicity and therapeutic efficacy of MTX during gut sterilization.

We thank Ms D. Alford Hoyt and Ms T. Gregorio for expert technical assistance and Ms A. Tvardzik and Ms C. Williams for assistance with the patient studies.

### REFERENCES

BODEY, G. P., GEHAN, E. A., FREIREICH, E. J. & FREI, E., III, (1971) Protected Environment—Prophylactic Antibiotic Program in the Chemotherapy of Acute Leukemia. *Am. J. Sci.*, **262**, 138.

COHEN, M. H., CREAVEN, P. J., FOSSIECK, B. E., JR., TVARDZIK, A. V. & WILLIAMS, C. L. (1975) Effect of Prophylactic Broad Spectrum Nonabsorbable Gastrointestinal Antibiotics (PNAA) on Absorption of Methotrexate and on other Metabolic Parameters of Lung Cancer Patients. *Proc Am. Soc. clin. Oncol.*, **16**, 154.

CREAVEN, P. J., HANSEN, H. H., ALFORD, D. A. & ALLEN, L. M. (1973) Methotrexate in Liver and Bile after Intravenous Dosage in Man. *Br. J. Cancer*, **28**, 589.

HENDERSON, E. S., ADAMSON, R. H. & OLIVERIO, V. T. (1965) The Metabolic Fate of Tritiated Methotrexate. II. Absorption and Excretion in Man. *Cancer Res.*, **25**, 1018.

HUFFMAN, D. H., WAN, S. H., AZAROFF, D. L. & HOOGSTRATEN, B. (1973) Pharmacokinetics of Methotrexate. *Clin. Pharmacol. Ther.*, **14**, 572.

KATES, R. E. & TOZER, T. N. (1973) Separation of Methotrexate and Nonmethotrexate Components in Rat Plasma (1973). *J. Pharm. Sci.*, **62**, 2056.

KNOTT, G. D. & REECE, D. K. (1972) MLAB: A Civilized Curve Fitting System. *Proc. On-Line*, **1**, 497.

KNOTT. G. D. & SCHRAGER, R. I. (1972) On-Line Modelling by Curve Fitting. *Proc. Siggraph.*, **6**, 138.

LEME, P. R., CREAVEN, P. J., ALLEN, L. M. & BERMAN, M. (1975) A Kinetic Model for the Disposition and Metabolism of Moderate and High Dose Methotrexate in Man. *Cancer Chemother. Rep.*, **59**, 811.

LEVINE, A. S., SIEGEL, S. E., SCHREIBER, A. D., HAUSER, J., PREISLER, H., GOLDSTEIN, I. M., SEIDLER, F., SIMON, R., PERRY, S., BENNET, J. E., & HENDERSON, E. S. (1973) Protected Environments and Prophylactic Antibiotics. A Prospective Controlled Study of Their Utility in the Therapy of Acute Leukemia. *New Engl. J. Med.*, **288**, 477.

VALERINO, D. M., JOHNS, D. G., ZAHARKO, D. S. & OLIVERIO, V. T. (1972) Studies of the Metabolism of Methotrexate by Intestinal Flora-I. *Biochem. Pharmacol.*, **21**, 821.

YATES, J. W. & HOLLAND, J. F. (1973) A Controlled Study of Isolation and Endogenous Microbial Suppression in Acute Myelocytic Leukemia Patients. *Cancer, N.Y.*, **32**, 1490.

ZAHARKO, D. S., BRUCKNER, H. & OLIVERIO, V. T. (1969) Antibiotics Alter Methotrexate Metabolism and Excretion. *Science, N.Y.*, **166**, 887.

ZAHARKO, D. S. & OLIVERIO, V. T. (1970) Reinvestigation of Methotrexate Metabolism in Rodents. *Biochem. Pharmac.*, **19**, 2923.